# Overexpression of Chromosome 21 miRNAs May Affect Mitochondrial Function in the Hearts of Down Syndrome Fetuses

**DOI:** 10.1155/2017/8737649

**Published:** 2017-09-05

**Authors:** Antonella Izzo, Rosanna Manco, Tiziana de Cristofaro, Ferdinando Bonfiglio, Rita Cicatiello, Nunzia Mollo, Marco De Martino, Rita Genesio, Mariastella Zannini, Anna Conti, Lucio Nitsch

**Affiliations:** ^1^Department of Molecular Medicine and Medical Biotechnology, University of Naples Federico II, 80131 Naples, Italy; ^2^Institute of Experimental Endocrinology and Oncology, National Research Council, 80131 Naples, Italy; ^3^Department of Biosciences and Nutrition, Karolinska Institutet, 17177 Stockholm, Sweden

## Abstract

Dosage-dependent upregulation of most of chromosome 21 (Hsa21) genes has been demonstrated in heart tissues of fetuses with Down syndrome (DS). Also miRNAs might play important roles in the cardiac phenotype as they are highly expressed in the heart and regulate cardiac development. Five Hsa21 miRNAs have been well studied in the past: miR-99a-5p, miR-125b-2-5p, let-7c-5p, miR-155-5p, and miR-802-5p but few information is available about their expression in trisomic tissues. In this study, we evaluated the expression of these miRNAs in heart tissues from DS fetuses, showing that miR-99a-5p, miR-155-5p, and let-7c-5p were overexpressed in trisomic hearts. To investigate their role, predicted targets were obtained from different databases and cross-validated using the gene expression profiling dataset we previously generated for fetal hearts. Eighty-five targets of let-7c-5p, 33 of miR-155-5p, and 10 of miR-99a-5p were expressed in fetal heart and downregulated in trisomic hearts. As nuclear encoded mitochondrial genes were found downregulated in trisomic hearts and mitochondrial dysfunction is a hallmark of DS phenotypes, we put special attention to let-7c-5p and miR-155-5p targets downregulated in DS fetal hearts and involved in mitochondrial function. The let-7c-5p predicted target *SLC25A4/ANT1* was identified as a possible candidate for both mitochondrial and cardiac anomalies.

## 1. Introduction

Down Syndrome (DS) is a major cause of congenital heart defects (CHD), mainly represented by atrioventricular canal defect (AVCD), ventricular septal defect (VSD), and tetralogy of Fallot (TOF) [1]. Most of them derive from the abnormal development of the endocardial cushions [1, 2]. Defects of the outflow tract are also frequent.

Attempts to identify chromosome 21 (Hsa21) genes possibly contributing to the DS phenotype have focused in the past on the Down syndrome critical region (DSCR). The DSCR hypothesis assumed that one or more genes in this region may be sufficient to produce the specific DS features when present in three copies [3].

A chromosome segment spanning from *D21S3* to *PFKL* in band 21q22.3 was considered a critical region for cardiac anomalies in DS (DS-CHD) [4]. Later, a 5.4 Mb genomic region was identified in the DS mouse model Dp (16) associated to congenital heart defects similar to that observed in DS subjects [5]. This region, which spans from *Tiam1* and *Kcnj6* and includes 52 Hsa21 ortholog genes, was further narrowed to 3.7 Mb [6] from *Ifnar1* and *Kcnj6* (35 Hsa21 ortholog genes). The two CHD critical regions described by Barlow and by Liu, identified according to different criteria, were mapped to very different loci of Hsa21. Discrepancies like this one suggest today that the origins of trisomic phenotypes are more complicated than formerly assumed and that they possibly involve multiple gene interactions.

Another interesting detail is that only about 50% of DS individuals manifest CHD. This means that we cannot hypothesize a simple correlation between gene overexpression and cardiac alterations. Therefore, a more extensive analysis of both transcriptome data and pathway perturbations must be applied to identify the complex molecular defects underlying CHD in DS.

In a previous study, we applied the microarray technology to analyze genome-wide expression profiles of the heart of DS fetuses with and without CHD [7]. The rationale for analyzing fetal tissues was based on the concept that CHD are thought to arise from anomalies in cardiac morphogenesis. By this approach, it was found that Hsa21 gene expression was globally upregulated 1.5 fold in trisomic samples, in general agreement with the gene dosage hypothesis. More than 400 genes located on other chromosomes were also differentially expressed, either upregulated or downregulated, between trisomic and nontrisomic hearts. Functional class scoring and gene set enrichment analyses of these genes revealed a global downregulation of nuclear-encoded mitochondrial genes (NEMGs) and upregulation of genes encoding extracellular matrix proteins. These data indicate that dosage-dependent upregulation of Hsa21 genes causes dysregulation of the genes responsible for mitochondrial function and for the extracellular matrix organization in the fetal heart of trisomic subjects and suggest that these alterations might be a prelude to heart defects. However, no significant differences in gene expression in hearts from DS fetuses (18–22 gestational weeks) with CHD could be found [7].

Throughout that study only the expression of protein-coding RNAs was analyzed even though noncoding RNAs, such as microRNAs (miRNAs), expressed in the heart, might be implicated in determining the CHD as they control protein expression in development, differentiation, and metabolism [8].

Recent findings indicate the involvement of microRNAs in mouse cardiac development and diseases [9–12]. In the context of DS, this can occur by 2 mechanisms: (i) Hsa21 miRNAs could be overexpressed as a consequence of the trisomy and could affect target genes directly or indirectly involved in heart morphogenesis, and (ii) Non-Hsa21 dysregulated miRNAs might affect target genes involved in heart morphogenesis.

In this study, we have investigated the former mechanism in heart tissues from DS fetuses with and without CHD.

Hsa21 encodes several classes of noncoding RNAs, the most enriched being long noncoding RNAs, while miRNAs are the less represented [13]. The most recent annotation of miRNA database (miRBase, release 21) has indicated that the Hsa21 harbors at least 29 miRNAs.

**Figure 1:**
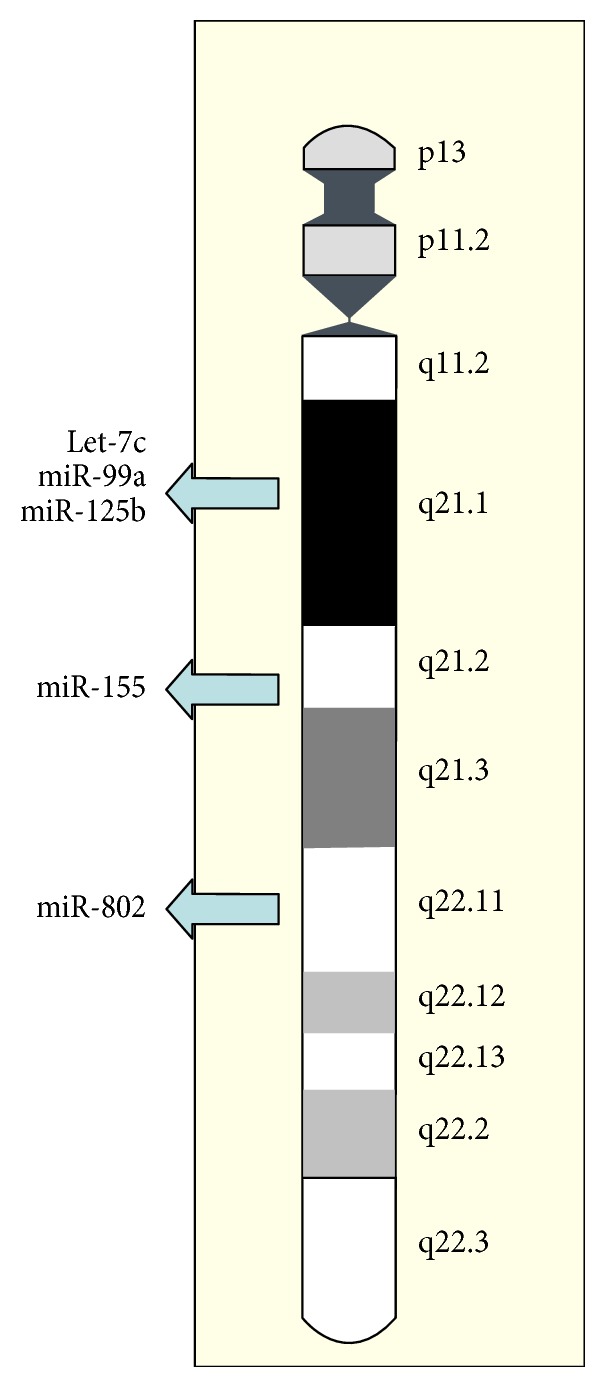
Hsa21 miRNAs. miR-99a-5p, let-7c-5p, and miR-125b-2-5p are located on Hsa21 in the sense orientation within an intron of the *C21orf34* gene, located at the beginning of q21.1 band. miR-99a-5p and let-7c-5p are only 659 bp apart, whereas miR-125b-2-5p lies just over 50.000 bp downstream of let-7c. miR-155-5p is located within the *BIC* gene, almost 9 Mb downstream from the *C21orf34* gene at Hsa21 genomic position q21.1. Finally, miR-802-5p is located just over 10 million bp downstream from the *BIC/miR-155* gene in the antisense orientation within intron 1 of the *RUNX1* gene at position q22.11.

Five of them, namely miR-99a-5p, let-7c-5p, miR-125b-2-5p, miR-155-5p, and miR-802-5p, were first identified (Figure 1). Their involvement in different DS associated phenotypes has been established, and several of their targets have been experimentally validated [14–20].

Few information is available about the expression of Hsa21-derived miRNAs in human heart tissues and their possible role in cardiomyogenesis.

We have analyzed the expression of 5 well-studied Hsa21 miRNAs to determine if they are dysregulated as consequence of trisomy and if their dysregulation might affect molecular mechanisms involved in mitochondrial function and heart development. To this aim, a bioinformatic analysis of Hsa21 miRNA target prediction by different databases was performed and cross-validated using the gene expression profiling dataset we previously generated for trisomic fetal hearts.

## 2. Materials and Methods

### 2.1. Samples

Cardiac tissues were obtained from fetuses at 18–22 weeks of gestation after therapeutic abortion according to protocols approved by our Institutional Ethical Committee. Tissues and RNAs were stored at the Telethon Bank of Fetal Biological Samples at the University of Naples. For this study, 3 hearts from euploid fetuses (NH), 3 from fetuses with DS without CHD (DH), and 3 from fetuses with DS and CHD (CDH) were analyzed. For let-7c mimic and inhibitor transfection, 2 previously characterized [21, 22] primary lines of euploid (N-HFF) and trisomic (DS-HFF) fetal fibroblasts were, respectively, used. Fibroblasts from biopsies were cultured in T25 flasks (BD Falcon) with Chang Medium B + C (Irvine Scientific) supplemented with 1% penicillin/streptomycin (Gibco) at 37°C in 5% CO_2_ atmosphere; all the analyses described throughout this study were carried out at cell culture passages 4-5.

### 2.2. RNA Extraction and Quantitative Real-Time PCR

Total RNA from each sample was extracted using TRIzol Reagent (Gibco/BRL Life Technologies Inc., Gaithersburg, MD) and was reverse-transcribed using iScript cDNA synthesis kit (Bio-Rad). qRT-PCR was performed using SsoAdvanced Universal SYBR Green Supermix (Bio-Rad) on a Bio-Rad iCycler CFX96 Touch Real-Time PCR Detection System according to the manufacturer's protocols. Primer pairs (MWG-Biotech, Ebersberg, Germany) were designed using the Primer 3 software (http://bioinfo.ut.ee/primer3-0.4.0/primer3; date last accessed 2015) to obtain amplicons ranging from 100 to 150 base pairs. In order to test primer efficiency, serial dilutions of cDNAs generated from selected samples, which expressed the target genes at a suitable level, were used to generate standard curves for each gene. qRT-PCR results are presented as relative mRNA levels normalized against reference control values. The GAPDH housekeeping gene was chosen as reference gene. Primer sequences of analyzed genes are the following *SLC25A4/ANT1*-F: GGGTTTCAACGTCTCTGTCC; *SLC25A4/ANT1*-R: TCCAGCTCACAAAAATGTGC; *DICER*-F: CTGATGGAATTAGAAGAAGCACTTAAT; *DICER*-R: ACCAGGGTCCCAGAACTACC; *GAPDH*-F: TGCACCACCAACTGCTTAGC; and *GAPDH ***-R:** GGCATGGACTGTGGTCATGAG.

For miRNA reverse transcription miScript II RT Kit (Qiagen) was used, and the mature miRNAs, mir-99a-5p, let-7c-5p, mir-125b-2-5p, mir-155-5p, and mir-802-5p, were quantified using miScript Primer Assay system and miScript SYBR Green PCR Kit (Qiagen). RNAUS and RNA5S were used as reference genes.

For end point PCR, assays were performed using the PCR Master Mix (2X) Kit (Thermo Fisher #K0171). Amplification products were visualized on 2% agarose gel and quantified using the Fiji software (http://www.fiji.sc) [23].

### 2.3. Bionformatic Analysis

Predicted miRNA-mRNA interactions were retrieved from 9 different algorithms (DIANA-microT [24], TargetScan [25], PITA [26], TargetMiner [27], miRDB [28], RNA22 [29], Pictar [30], and MiRanda [31]) using as input the sequences/IDs for the miRNA of interest. Before merging the date, divergent database annotations were unified by converting transcript-wise predictions to the gene level (Ensembl gene ID) using the BiomaRt package [32]. The lists of targets predicted by each database were subsequently marked with Affymetrix probe IDs using the related annotation package and finally merged with the list of probes (*n* = 279) found significantly downregulated in DS hearts compared to euploid controls [7]. For further analyses, we brought forward miR targets predicted by at least two databases, with a *P* value ≤ 0.05 and fold change ≤ −1.2 as defined in the original paper [7].

Gene ontology (GO) functional class scoring of the lists of significantly downregulated genes was performed using the Web-based GEne SeT Analysis Toolkit V2 (http://www.webgestalt.org) [33]. Special attention was given to mitochondria-related categories and pathways.

### 2.4. miRNA Mimic Transfection

For let-7c-5p upregulation, a let-7c miRNA mimic (miScript miRNA Mimics, Qiagen) was transfected in 2 N-HFF lines. Cells were plated in a concentration of 70,000/well on 24 well plates (BD Falcon) and after 24 hours were transfected with a miRNA mimic using the INTERFERin transfection reagent (Polyplus-transfection). A fluorescent siRNA (AllStars Neg. siRNA AF 488, Qiagen) has been used to monitor the efficiency of the chosen transfecting agent. Forty-eight hours after the transfection cells were harvested and *ANT1* and *DICER* expression were evaluated, cells treated with the INTERFERin transfection agent only were used as mock control for all experiments performed after transfection.

### 2.5. miRNA Inhibitor Transfection

For let-7c-5p downregulation, an Anti-hsa-let-7c miRNA (miScript miRNA Inhibitor, Qiagen) was transfected in 2 DS-HFF lines. Cells were plated in a concentration of 150,000/dish on 3,5 cm petri dishes (BD Falcon) and after 24 hours were transfected with the inhibitor using the INTERFERin transfection reagent (Polyplus-transfection). Forty-eight hours after transfection cells were harvested for RNA collection while 72 hours after transfection protein lysates were obtained.

### 2.6. Immunoblot Analysis

Cells were lysed in radioimmunoprecipitation assay buffer (1% Triton; 0,5% sodium deoxycholate; 0,1% sodium dodecyl sulfate; 0,15 M NaCl; 0,05 tris-HCl; and pH 7.2) supplemented with protease inhibitors. For western blot analysis, proteins were separated on SDS-PAGE (Mini-Protean TGX gels, Bio-Rad), gels were blotted onto Immobilon-P (Millipore, Bedford, MA, USA) for overnight, and the membranes were blocked in 5% nonfat dry milk in PBS plus 0,05% Tween 20 for 2 h or overnight before the addition of the primary antibody for 2 h. The primary antibodies used were anti-*SLC25A4/ANT1* mAb (ab 110322, Abcam) and anti-vinculin (N-19) (sc-7649, Santa Cruz).

The filters were washed three times in PBS plus 0.05% Tween 20 before the addition of horseradish peroxidase-conjugated secondary antibodies for 45 min. Horseradish peroxidase was detected with ECL (Pierce).

### 2.7. Statistics

The Student's *t*-test was applied to evaluate the statistical significance of data in this study. The threshold for statistical significance (*P* value) was set at 0.05.

## 3. Results

We evaluated, by qRT-PCR, the expression of Hsa21 miRNAs in fetal heart samples from 6 DS individuals, 3 with CHD (CHD samples) and 3 without CHD (DH samples), and from 3 nontrisomic controls (NH samples).

**Figure 2:**
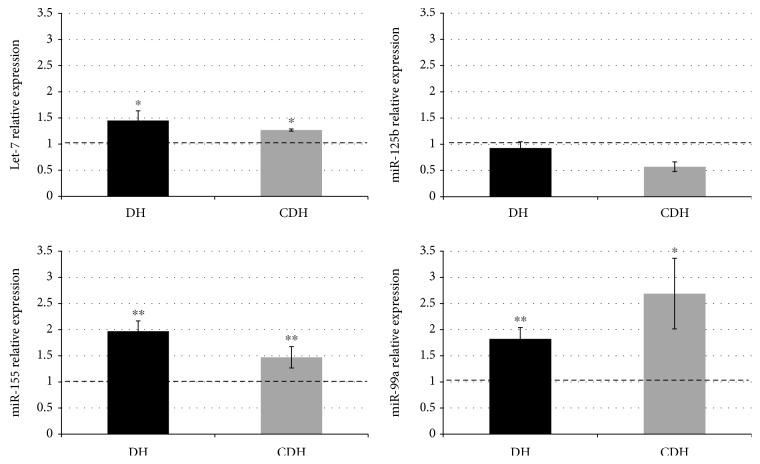
Hsa21 miRNA expression evaluated by qRT-PCR in fetal hearts. Let-7c-5p, miR-99a-5p, and miR-155-5p were upregulated in trisomic hearts without cardiopathy (DH) and with cardiopathy (CDH). miR-125b-2-5p was variably expressed and not upregulated. Results are expressed as relative mean values ± SEM of 3 DH and 3 CDH samples, compared with control hearts (NH) set equal to 1. ^∗^*P* < 0.05, ^∗∗^*P* < 0.001. *P* value expresses statistical significance for trisomic versus nontrisomic sample comparisons.

We found that miR-99a-5p, miR-125b-2-5p, let-7c-5p, and miR-155-5p were expressed in fetal hearts at 18–22 gestational weeks. miR-802-5p was not expressed. miR-99a-5p, miR-155-5p, and let-7c-5p were overexpressed in trisomic hearts when compared with euploid ones, whereas miR-125b-2-5p was not dysregulated and was quite variably expressed (Figure 2). The comparison between heart tissues from fetuses with and without CHD indicated that miR-155-5p and let-7c-5p did not show significant differences between DH and CDH samples while miR-99a-5p was differentially expressed even though the sample size is too small to obtain a statistical significance.

As miRNAs could affect protein expression by either interfering with RNA translation or promoting mRNA degradation [34], we looked at mRNA expression of target genes of overexpressed miRNAs by using the dataset of our previous study by which we investigated gene expression profiling in the same hearts [7].

We considered as target list the sum of all targets predicted by 9 different software from miRBase annotation, and we filtered them according to gene expression microarray results. The rationale of our approach is based on the notion that overexpression of Hsa21 miRNAs can result in downregulation of specific target genes possibly involved in DS phenotype. For each miRNA, we compared target genes predicted at least by 2 different software with the list of genes downregulated in trisomic hearts with a fold change (FC) > |1,2| and *P* value< 0.05. Using these criteria, we unraveled that 85 targets of let-7c-5p (Supplementary Table 1 available online at https://doi.org/10.1155/2017/8737649), 33 of miR-155-5p (Supplementary Table 2), and 10 of miR-99a-5p (Supplementary Table 3) were expressed in fetal heart and downregulated in trisomic samples.

Over-representation analysis (ORA) of these lists, performed using the Web-based GEne SeT Analysis Toolkit (http://www.webgestalt.org) [33], demonstrated a highly significant enrichment (*P* < 0.0001) of the cell component gene ontology category “mitochondrion” for let-7c-5p and miR-155-5p downregulated targets, with a cluster of 26 and 8 genes, respectively (Tables 1 and 2). No mitochondria-related genes were found among targets of miR-99a-5p.

We focused our attention on the *SLC25A4/ANT1* gene (indicated in bold in Table 1), which was predicted by 5 databases as a let-7c-5p target gene and already proposed as a target of both human and murine let-7b [35–37]. Its downregulation in heart tissue was confirmed by qRT-PCR (Supplementary Figure 1). According to the STarMirDB, a database of microRNA binding sites based on cross-linking immunoprecipitation (CLIP) data (http://sfold.wadsworth.org/starmirDB.php) [38], seven binding sites for let-7c-5p are located in the *SLC25A4/ANT1* sequence. One of them has been mapped to a conserved 3'UTR seed site (seed type 7mer-A1) [39] with a logistic probability of 0.68.

**Figure 3:**
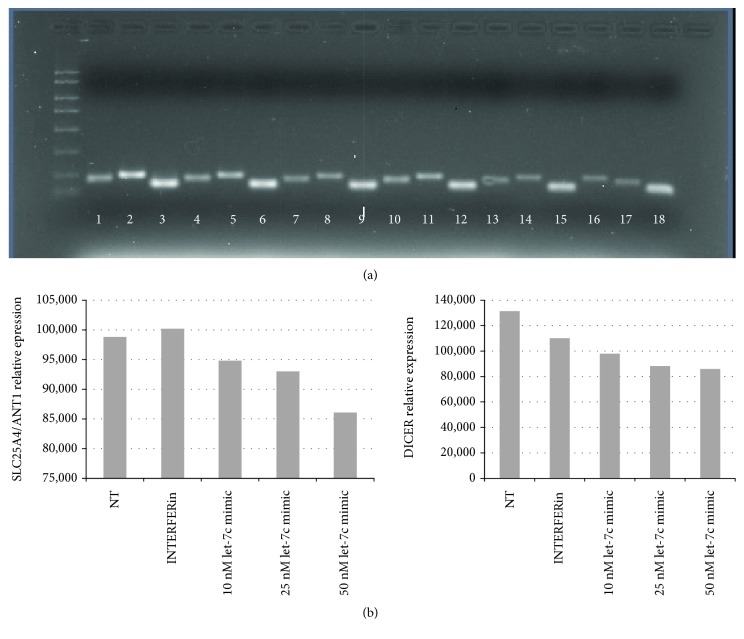
End point PCR of *SLC25A4/ANT1*, *DICER*, and *GAPDH* in euploid fibroblasts transfected with a let-7c-5p mimic. (a) Agarose gel of *SLC25A4/ANT1*, *DICER*, and *GAPDH* amplification in: Not transfected cells (lanes 1–3); cells + INTERFERin (lanes 4–6); cells + 5 nM miRNA mimic (lanes 7–9); cells + 10 nM miRNA mimic (lanes 10–12); cells + 25 nM miRNA mimic (lanes 13–15); cells + 50 nM miRNA mimic (lanes 16–18). Amplification products of *SLC25A4/ANT1*: lanes 1, 4, 7, 10, 13, and 16; amplification products of *DICER*: lanes 2, 5, 8, 11, 14, and 17; amplification products of *GAPDH*: lanes 3, 6, 9, 12, 15, and 18. (b) Densitometric analysis of *SLC25A4/ANT1* and *DICER* amplification products obtained by ImageJ software. At 25 nM miRNA mimic concentration, a reduction of target amplification is appreciable.

We were interested in this gene because of its central role in oxidative phosphorylation (OXPHOS). Our hypothesis was that the *SLC25A4/ANT1* downregulation could be an effect of the specific interaction between let-7c-5p and this gene at the seed sequence. To validate this hypothesis, we performed a transfection experiment of a let-7c-5p mimic miRNA in euploid fibroblasts in which *SLC25A4/ANT1* is expressed. As control, we evaluated also the expression of *DICER*, since it is a let-7c-5p validated target [40]. We transfected fibroblasts at different concentrations of miRNA mimic for 48 h, and we measured *SLC25A4/ANT1*, *DICER*, and *GAPDH* expression by end point PCR. As shown in Figure 3, at 25 nM miRNA dosage (lanes 13-14), a reduction of target amplification is already appreciable with respect to nontransfected cells (lanes 1-2) or cells transfected with transfection reagent only (lanes 4-5). As expected, *GAPDH* amplification did not show variations.

**Figure 4:**
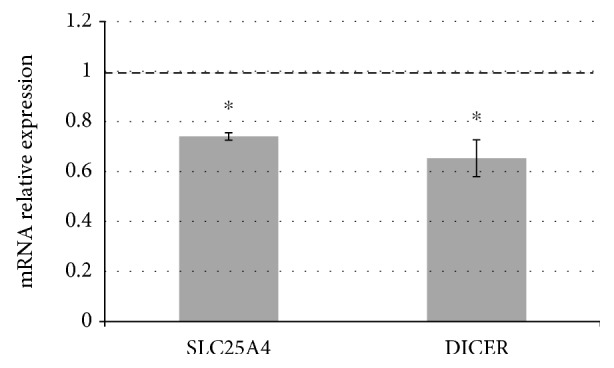
*SLC25A4/ANT1* and *DICER* expression by qRT-PCR. Transfection of let-7c-5p mimic induces a significant decrease of *SLC25A4/ANT1* and *DICER* expression in euploid fibroblasts. Results are expressed as relative mean values ± SEM of three different determination, compared with cells treated only with transfecting agent (set equal to 1). ^∗^*P* < 0.05.

We further evaluated *SLC25A4/ANT1* and *DICER* expression by qRT-PCR showing that after let-7c mimic transfection (25 nM), their expression significantly decreased if compared with cells transfected with the transfecting agent only (Figure 4).

**Figure 5:**
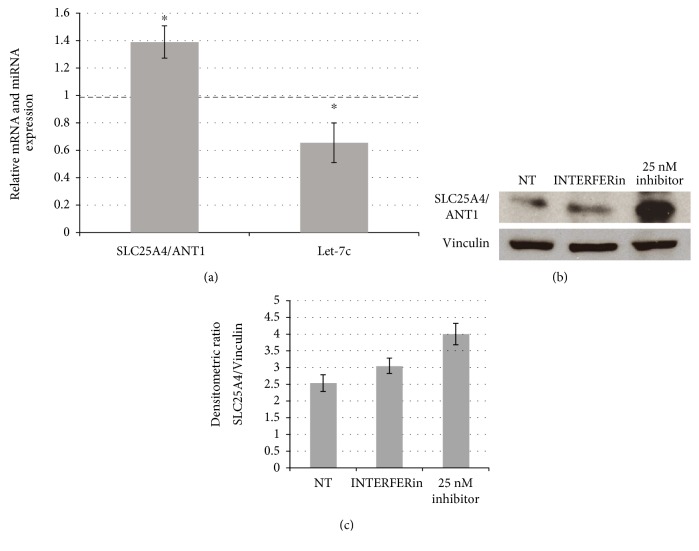
*SLC25A4/ANT1* after let-7c-5p inhibition in trisomic fibroblasts. (a) Transfection of let-7c-5p inhibitor in DS-HFFs induces a significant increase of *SLC25A4/ANT1* expression and a decrease of let-7c-5p, as detected by qRT-PCR. Results are expressed as relative mean values ± SEM of three different determination, compared with cells treated only with transfecting agent (set equal to 1). (b) Representative immunoblot of SLC25A4/ANT1 in nontransfected cells (NT), cells transfected only with transfecting agent and cells transfected with 25 nM let-7c-5p inhibitor. Vinculin was used as loading control. (c) Densitometric SLC25A4/ANT1 relative measurement after 72 h treatment. Protein expression was significantly increased after inhibiting treatment if compared with mock transfected cells. The bars show relative mean values ± SEM of two DS-HFFs in duplicate; ^∗^*P* < 0.05.

Finally, we performed the reciprocal experiment using a let-7c-5p inhibitor in trisomic fibroblasts collecting both RNA and proteins. After treatment with 25 nM inhibitor, which reduced let-7c-5p expression by 35%, the expression of *SLC25A4/ANT1* was significantly reduced both at mRNA (a) and protein (b-c) level (Figure 5).

## 4. Discussion

We have analyzed the expression of 5 Hsa21 miRNAs in trisomic fetal hearts at 18–22 gestational weeks to investigate whether they are affected by the gene dosage effect observed for most of the Hsa21 genes. Three out of five, namely let-7c-5p, miR-99a-5p, and miR-155-5p, were significantly overexpressed, if compared with euploid controls. The upregulation was not higher than 2.5 folds in agreement with a gene dosage effect. In the same samples, miR-802-5p was not expressed and miR-125b-2-5p was normoregulated. This was not surprising as the miR-125b-1-5p, which maps to 11q24.1, encoding an identical miRNA, may mask the eventual overexpression.

Comparison between hearts from fetuses with cardiopathies versus hearts from fetuses without cardiopathies showed that miR-99a-5p was more expressed in the latter samples even though more samples are needed to reach a significant result due to a high variability.

In a recent paper, the role of mmu-let-7c and mmu-miR-99a in cardiomyogenesis was investigated using an overexpression strategy in murine embryonic stem cells. By overexpressing these miRNAs at early stage of differentiation, the authors demonstrated that they are involved in heart development as let-7c induces cardiogenesis while miR-99a appears to repress it by altering the *Smad2* signaling [18].

The overexpression of Hsa21 miRNAs can result in downregulation of specific target genes possibly involved in DS phenotype. To select relevant miRNA targets we have used the following criteria:

- Bioinformatics prediction
- mRNA decreased expression in heart samples from DS fetuses
- Inclusion in the category of mitochondria related genes
- Involvement in normal and/or abnormal heart development

Among the selected genes, *SLC25A4/ANT1*, a predicted let-7c-5p target downregulated in trisomic hearts, appeared as a potential candidate for both mitochondrial dysfunction and CHD in DS. *SLC25A4* (Solute carrier family 25 member 4) or *ANT1* (Adenine nucleotide translocator 1) functions as a gated pore that translocates ADP and ATP between cytoplasm and mitochondria, regulating the intracellular energetic balance. Furthermore, its dysregulation has been associated to mitochondrial cardiomyopathies [41].

Humans have four *ANT* isoforms that are encoded by four different genes and are distributed in a tissue specific pattern. The human SLC25A4/*ANT1* gene is primarily expressed in the heart and in the skeletal muscle [42]. It is downregulated in DS fetal hearts and fibroblasts in which let-7c-5p is upregulated. qRT-PCR after let-7c-5p overexpression showed an inverse relationship with *SLC25A4/ANT1*, which was downregulated. The reverse experiment using miRNA inhibitor in trisomic cells demonstrated a significant increase of RNA and protein expression of the gene while let-7c-5p expression was decreased if compared with mock controls. These results may be considered an additional experimental support of our hypothesis even though further experiments will be necessary to definitely validate that *SLC25A4/ANT1* is a let-7c-5p target, which is beyond the purpose of this paper. To this aim, we plan to perform a luciferase assay to demonstrate a direct interaction.

OXPHOS deficiency and mitochondrial dysfunction have been associated with developmental mechanisms of DS [43]. *SLC25A4/ANT1* is one of the 37 NEMGs downregulated after *NRIP1* overexpression in the GEO GSE 19836 experiment [38] that we reanalyzed in Izzo et al. [22]. After *NRIP1* silencing in trisomic cells [23], *SLC25A4/ANT1* expression increased, thus allowing a more efficient exchange of ATP and an improvement of mitochondrial activity in DS samples. Accordingly, genetic inactivation of the heart isoform results in mtDNA damage and increased reactive oxygen species [44].

The above described strategy to select targets relevant for mitochondrial function and heart development was applied also to the miR-155-5p. Thirty-three predicted target genes of miR-155-5p have been found downregulated in trisomic fetal hearts if compared with euploid ones. Eight of them, including *MECP2* gene, which is a validated target [37, 45], are included in the cell component GO category “mitochondrion” suggesting an impact of this miRNA on mitochondrial function. *MECP2* is included also among the let-7c-5p predicted targets.

It was recently reported that the Hsa21 miR-155-5p regulates mitochondrial biogenesis by targeting mitochondrial transcription factor A (*TFAM*) [46]. Surprisingly, after studying both DS fibroblasts and heart samples, the authors conclude that the regulation of *TFAM* by the miRNA impacts mitochondrial biogenesis in the euploid setting but not in the DS setting. We suggest that a possible explanation may lie in the fact that mitochondrial biogenesis is already so decreased in DS cells to mask further defects. *TFAM* was not identified by our strategy because its heart expression at 18–22 gestational weeks is too low to be analyzed by microarray technology.

Finally miR-155-5p has been hypothesized to be an inducer of cardiac hypertrophy [47]. Its inhibition might have clinical potential to counteract this pathology.

## 5. Conclusions

In this study, we demonstrated the overexpression of three Hsa21 miRNAs, miR-99a-5p, miR-155-5p, and let-7c-5p, in the heart of trisomic fetuses as a likely consequence of gene dosage effect. Bioinformatic analysis demonstrated that two of these miRNAs, let-7c-5p and miR-155-5p, count several predicted targets among genes involved in mitochondrial function that were found to be downregulated in trisomic fetal hearts. Among the let-7c predicted targets, we identified and partially validated *SLC25A4/ANT1*, a gene encoding the main translocator of ADP/ATP across the mitochondrial membrane, which plays a major role in mitochondrial function.

Our results also support the hypothesis that the overexpression of miR-155-5p might have a potential impact on mitochondrial biogenesis.

Both miRNAs, let-7c-5p and miR-155-5p, should be taken into account while investigating the molecular mechanisms causing cardiac malformations in DS.

## Supplementary Material

Table 1. Targets of let-7c significantly downregulated in DS fetal hearts with a (FC)>|1,2| and p-value<0,05. Number of predictions by different databases are also indicated. Table 2. Targets of miR-155 significantly downregulated in DS fetal hearts with a (FC)>|1,2| and p-value<0,05. Number of predictions by different databases are also indicated. Table 3. Targets of miR-99a significantly downregulated in DS fetal hearts with a (FC)>|1,2| and p-value<0,05. Number of predictions by different databases are also indicated. Figure 1. *SLC25A4/ANT1* expression in heart tissue by qRT-PCR. Real-time PCR of SLC25A4/ANT1 in trisomic hearts confirmed the downregulation obtained by microarray analysis. Results are expressed as relative mean values ± SEM of 3 trisomic samples (DSH), compared with control hearts (NH) set equal to 1. ∗∗ p <0.01. P-value expresses statistical significance for trisomic versus non trisomic sample comparisons.

## Figures and Tables

**Table 1:** Targets of let-7c-5p significantly downregulated in DS hearts and belonging to gene ontology cellular component category “mitochondrion.” Twenty-six genes were observed instead of the expected 6.49 genes with *P* < 0.00001.

Gene symbol	Description	FC
AKAP8	A-kinase anchoring protein 8	−1,361
COX10	COX10, heme A: farnesyltransferase cytochrome c oxidase assembly factor	−1,307
COX5A	Cytochrome c oxidase subunit 5A	−1,263
DLAT	Dihydrolipoamide S-acetyltransferase	−1,678
DLST	Dihydrolipoamide S-succinyltransferase	−1,724
FKBP4	FK506 binding protein 4	−1,558
GADD45GIP1	GADD45G interacting protein 1	−1,664
GHITM	Growth hormone inducible transmembrane protein	−1,477
HSPB7	Heat shock protein family B (small) member 7	−1,422
MECP2	Methyl-CpG binding protein 2	−1,22
MPC1	Mitochondrial pyruvate carrier 1	−1,339
MRPL33	Mitochondrial ribosomal protein L33	−1,333
MRS2	MRS2, magnesium transporter	−1,416
NDUFS3	NADH: ubiquinone oxidoreductase core subunit S3	−1,314
NMT1	N-myristoyltransferase 1	−1,443
NOL7	Nucleolar protein 7	−1,299
PANK2	Pantothenate kinase 2	−1,361
PCCB	Popionyl-CoA carboxylase beta subunit	−1,335
PDHA1	Pyruvate dehydrogenase (lipoamide) alpha 1	−1,524
PGS1	Phosphatidylglycerophosphate synthase 1	−1,233
SDHC	Succinate dehydrogenase complex subunit C	−1,23
SLC25A12	Solute carrier family 25 member 12	−1,408
**SLC25A4**	Solute carrier family 25 member 4	**−1,55**
TIMM23	Translocase of inner mitochondrial membrane 23	−1,508
UQCC1	Ubiquinol-cytochrome c reductase complex assembly factor 1	−1,517
UQCRFS1	Ubiquinol-cytochrome c reductase, Rieske iron-sulfur polypeptide 1	−1,292

**Table 2:** Targets of miR-155-5p significantly downregulated in DS hearts and belonging to gene ontology cellular component category “mitochondrion.” Eight genes were observed instead of the expected 2.34 genes with *P* < 0.00001.

Gene Symbol	Description	FC
DLAT	Dihydrolipoamide S-acetyltransferase	−1,678
HSPB7	Heat shock protein family B (small) member 7	−1,422
LPIN1	Lipin 1	−1,838
MECP2	Methyl-CpG binding protein 2	−1,22
NDUFS3	NADH: ubiquinone oxidoreductase core subunit S3	−1,314
NT5C	5′, 3′-nucleotidase, cytosolic	−1,524
SIRT5	Sirtuin 5	−1,255
YWHAQ	Tyrosine 3-monooxygenase/tryptophan 5-monooxygenase activation protein theta	−1,364
