# Supplementary material for: Overexpression of Chromosome 21 miRNAs May Affect Mitochondrial Function in the Hearts of Down Syndrome Fetuses
Source: Int J Genomics. 2017 Sep 5;2017:8737649. doi: 10.1155/2017/8737649 (PMC5605795; doi:10.1155/2017/8737649)
Supplement: Supplementary file 2 [file 8737649.f2.docx]

**Table 1. Targets of let-7c significantly downregulated in DS fetal hearts with a (FC)>|1,2| and p-value<0,05. Number of predictions by different databases are also indicated.**

| **Gene Symbol** | **Description** | **FC** | **p-value** | **N. of predictions** |
| --- | --- | --- | --- | --- |
| E2F6 | E2F transcription factor 6 | -1,592 | 0,0328 | 6 |
| DLSTP1 | dihydrolipoamide S-succinyltransferase pseudogene 1 | -1,724 | 0,0138 | 5 |
| HIF1AN | hypoxia inducible factor 1 alpha subunit inhibitor | -1,575 | 0,0093 | 5 |
| SLC25A12 | solute carrier family 25 member 12 | -1,408 | 0,0088 | 5 |
| SLC25A4 | solute carrier family 25 member 4 | -1,55 | 0,0263 | 5 |
| ARMC8 | armadillo repeat containing 8 | -1,541 | 0,0463 | 4 |
| H2AFV | H2A histone family member V | -1,259 | 0,0039 | 4 |
| MRS2 | MRS2, magnesium transporter | -1,416 | 0,0054 | 4 |
| PRDM2 | PR/SET domain 2 | -1,221 | 0,0321 | 4 |
| RBPMS | RNA binding protein with multiple splicing | -1,427 | 0,0046 | 4 |
| SORBS2 | sorbin and SH3 domain containing 2 | -1,451 | 0,0364 | 4 |
| ABI2 | abl interactor 2 | -1,253 | 0,0137 | 3 |
| AKAP8 | A-kinase anchoring protein 8 | -1,361 | 0,0104 | 3 |
| ALPK3 | alpha kinase 3 | -1,406 | 0,0207 | 3 |
| AREL1 | apoptosis resistant E3 ubiquitin protein ligase 1 | -1,429 | 0,0361 | 3 |
| ARPC5L | actin related protein 2/3 complex subunit 5 like | -1,351 | 0,0333 | 3 |
| ASPH | aspartate beta-hydroxylase | -1,403 | 0,0468 | 3 |
| CLK2 | CDC like kinase 2 | -1,422 | 0,0385 | 3 |
| EFEMP1 | EGF containing fibulin like extracellular matrix protein 1 | -1,453 | 0,0268 | 3 |
| GLRX3 | glutaredoxin 3 | -1,232 | 0,0384 | 3 |
| HSPB7 | heat shock protein family B (small) member 7 | -1,422 | 0,032 | 3 |
| KDM5C | lysine demethylase 5C | -1,404 | 0,043 | 3 |
| MAX | MYC associated factor X | -1,25 | 0,0485 | 3 |
| MECP2 | methyl-CpG binding protein 2 | -1,22 | 0,0381 | 3 |
| NUDT15 | nudix hydrolase 15 | -1,342 | 0,0318 | 3 |
| PANK2 | pantothenate kinase 2 | -1,361 | 0,0395 | 3 |
| PCDH7 | protocadherin 7 | -1,245 | 0,0261 | 3 |
| PDLIM5 | PDZ and LIM domain 5 | -1,456 | 0,0265 | 3 |
| TIMM23 | translocase of inner mitochondrial membrane 23 | -1,508 | 0,0382 | 3 |
| TROVE2 | TROVE domain family member 2 | -1,248 | 0,0414 | 3 |
| UBE2G1 | ubiquitin conjugating enzyme E2 G1 | -1,299 | 0,0465 | 3 |
| WBP4 | WW domain binding protein 4 | -1,517 | 0,0508 | 3 |
| YLPM1 | YLP motif containing 1 | -1,364 | 0,0239 | 3 |
| ZNF219 | zinc finger protein 219 | -1,453 | 0,0206 | 3 |
| APOLD1 | apolipoprotein L domain containing 1 | -1,511 | 0,0036 | 2 |
| BLCAP | bladder cancer associated protein | -1,399 | 0,0022 | 2 |
| COX10 | COX10, heme A:farnesyltransferase cytochrome c oxidase assembly factor | -1,307 | 0,0101 | 2 |
| COX5A | cytochrome c oxidase subunit 5A | -1,263 | 0,04 | 2 |
| DIDO1 | death inducer-obliterator 1 | -1,481 | 0,0056 | 2 |
| DLAT | dihydrolipoamide S-acetyltransferase | -1,678 | 0,0377 | 2 |
| DNPEP | aspartyl aminopeptidase | -1,287 | 0,023 | 2 |
| DPYSL4 | dihydropyrimidinase like 4 | -2,404 | 0,0131 | 2 |
| EML5 | echinoderm microtubule associated protein like 5 | -1,597 | 0,0522 | 2 |
| FABP3 | fatty acid binding protein 3 | -1,692 | 0,017 | 2 |
| FKBP4 | FK506 binding protein 4 | -1,558 | 0,0549 | 2 |
| GADD45GIP1 | GADD45G interacting protein 1 | -1,664 | 0,0402 | 2 |
| GHITM | growth hormone inducible transmembrane protein | -1,477 | 0,0233 | 2 |
| HERPUD1 | homocysteine inducible ER protein with ubiquitin like domain 1 | -1,565 | 0,0331 | 2 |
| HLA-DRB1 | major histocompatibility complex, class II, DR beta 1 | -1,634 | 0,0069 | 2 |
| KPNB1 | karyopherin subunit beta 1 | -1,988 | 0,0012 | 2 |
| KRT8 | keratin 8 | -1,629 | 0,047 | 2 |
| MAT2A | methionine adenosyltransferase 2A | -1,621 | 0,0417 | 2 |
| METAP1 | methionyl aminopeptidase 1 | -1,381 | 0,0455 | 2 |
| MPC1 | mitochondrial pyruvate carrier 1 | -1,339 | 0,0129 | 2 |
| MRPL33 | mitochondrial ribosomal protein L33 | -1,333 | 0,0318 | 2 |
| MTMR9 | myotubularin related protein 9 | -1,626 | 0,0287 | 2 |
| NDST1 | N-deacetylase and N-sulfotransferase 1 | -1,307 | 0,0371 | 2 |
| NDUFS3 | NADH:ubiquinone oxidoreductase core subunit S3 | -1,314 | 0,0294 | 2 |
| NELFB | negative elongation factor complex member B | -1,316 | 0,0107 | 2 |
| NMT1 | N-myristoyltransferase 1 | -1,443 | 0,0081 | 2 |
| NOL7 | nucleolar protein 7 | -1,299 | 0,0258 | 2 |
| NQO2 | N-ribosyldihydronicotinamide:quinone reductase 2 | -1,348 | 0,0216 | 2 |
| PCCB | propionyl-CoA carboxylase beta subunit | -1,335 | 0,0462 | 2 |
| PDE4DIP | phosphodiesterase 4D interacting protein | -1,605 | 0,0172 | 2 |
| PDHA1 | pyruvate dehydrogenase (lipoamide) alpha 1 | -1,524 | 0,0088 | 2 |
| PDXDC1 | pyridoxal dependent decarboxylase domain containing 1 | -1,271 | 0,0363 | 2 |
| PGS1 | phosphatidylglycerophosphate synthase 1 | -1,233 | 0,0387 | 2 |
| PPFIA4 | PTPRF interacting protein alpha 4 | -1,629 | 0,0484 | 2 |
| RAB27A | RAB27A, member RAS oncogene family | -1,233 | 0,0186 | 2 |
| RAB40B | RAB40B, member RAS oncogene family | -1,475 | 0,0221 | 2 |
| RASL11B | RAS like family 11 member B | -1,473 | 0,0287 | 2 |
| RHBDF1 | rhomboid 5 homolog 1 | -1,269 | 0,0159 | 2 |
| SCHIP1 | schwannomin interacting protein 1 | -1,267 | 0,0381 | 2 |
| SDHC | succinate dehydrogenase complex subunit C | -1,23 | 0,0456 | 2 |
| SRSF5 | serine and arginine rich splicing factor 5 | -1,613 | 0,0541 | 2 |
| SUPT20H | SPT20 homolog, SAGA complex component | -1,529 | 0,0079 | 2 |
| TMEM161A | transmembrane protein 161A | -1,282 | 0,0414 | 2 |
| TMEM43 | transmembrane protein 43 | -1,252 | 0,0273 | 2 |
| TRIP10 | thyroid hormone receptor interactor 10 | -1,353 | 0,0476 | 2 |
| UBN1 | ubinuclein 1 | -1,357 | 0,0384 | 2 |
| UQCC1 | ubiquinol-cytochrome c reductase complex assembly factor 1 | -1,517 | 0,0119 | 2 |
| UQCRFS1 | ubiquinol-cytochrome c reductase, Rieske iron-sulfur polypeptide 1 | -1,292 | 0,0242 | 2 |
| USP13 | ubiquitin specific peptidase 13 (isopeptidase T-3) | -1,431 | 0,0317 | 2 |
| ZNF384 | zinc finger protein 384 | -1,215 | 0,0322 | 2 |
| ZNF672 | zinc finger protein 672 | -1,305 | 0,0173 | 2 |

**Table 2. Targets of miR-155 significantly downregulated in DS fetal hearts with a (FC)>|1,2| and p-value<0,05. Number of predictions by different databases are also indicated.**

| **Gene Symbol** | **Description** | **FC** | **p-value** | **N. of predictions** |
| --- | --- | --- | --- | --- |
| TRIM44 | tripartite motif containing 44 | -1,664 | 0,0429 | 5 |
| WEE1 | WEE1 G2 checkpoint kinase | -1,493 | 0,053 | 5 |
| ABI2 | abl interactor 2 | -1,253 | 0,0137 | 4 |
| ARMC8 | armadillo repeat containing 8 | -1,541 | 0,0463 | 4 |
| HIPK1 | homeodomain interacting protein kinase 1 | -1,401 | 0,052 | 4 |
| PDLIM5 | PDZ and LIM domain 5 | -1,456 | 0,0265 | 4 |
| EHD1 | EH domain containing 1 | -1,488 | 0,0118 | 3 |
| PCDH7 | protocadherin 7 | -1,245 | 0,0261 | 3 |
| PRDM2 | PR/SET domain 2 | -1,221 | 0,0321 | 3 |
| RANBP10 | RAN binding protein 10 | -1,311 | 0,0145 | 3 |
| RBAK, NT5C | RB associated KRAB zinc finger | -1,524 | 0,0288 | 3 |
| WBP4 | WW domain binding protein 4 | -1,517 | 0,0508 | 3 |
| ZNF91 | zinc finger protein 91 | -1,49 | 0,0103 | 3 |
| AATF | apoptosis antagonizing transcription factor | -1,23 | 0,0168 | 2 |
| ALPK3 | alpha kinase 3 | -1,406 | 0,0207 | 2 |
| DLAT | dihydrolipoamide S-acetyltransferase | -1,678 | 0,0377 | 2 |
| DUSP14 | dual specificity phosphatase 14 | -1,645 | 0,0128 | 2 |
| EEF1D | eukaryotic translation elongation factor 1 delta | -1,321 | 0,0475 | 2 |
| EML5 | echinoderm microtubule associated protein like 5 | -1,597 | 0,0522 | 2 |
| HIF1AN | hypoxia inducible factor 1 alpha subunit inhibitor | -1,575 | 0,0093 | 2 |
| HSPB7 | heat shock protein family B (small) member 7 | -1,422 | 0,032 | 2 |
| LPIN1 | lipin 1 | -1,838 | 0,0317 | 2 |
| MECP2 | methyl-CpG binding protein 2 | -1,22 | 0,0381 | 2 |
| MNAT1 | MNAT1, CDK activating kinase assembly factor | -1,383 | 0,0308 | 2 |
| NDUFS3 | NADH:ubiquinone oxidoreductase core subunit S3 | -1,314 | 0,0294 | 2 |
| NGRN | neugrin, neurite outgrowth associated | -1,342 | 0,0363 | 2 |
| RNF216 | ring finger protein 216 | -1,506 | 0,0185 | 2 |
| SCHIP1 | schwannomin interacting protein 1 | -1,267 | 0,0381 | 2 |
| SIRT5 | sirtuin 5 | -1,255 | 0,029 | 2 |
| SORBS2 | sorbin and SH3 domain containing 2 | -1,451 | 0,0364 | 2 |
| SUPT20H | SPT20 homolog, SAGA complex component | -1,529 | 0,0079 | 2 |
| TAX1BP1 | Tax1 binding protein 1 | -1,374 | 0,03 | 2 |
| YWHAQ | tyrosine 3-monooxygenase/tryptophan 5-monooxygenase activation protein theta | -1,364 | 0,0483 | 2 |

**Table 3.** **Targets of miR-99a significantly downregulated in DS fetal hearts with a (FC)>|1,2| and p-value<0,05. Number of predictions by different databases are also indicated.**

| **Gene Symbol** | **Description** | **FC** | **p-value** | **N. of predictions** |
| --- | --- | --- | --- | --- |
| HIPK1 | homeodomain interacting protein kinase 1 | -1,401 | 0,05 | 3 |
| ARPC5L | actin related protein 2/3 complex subunit 5 like | -1,351 | 0,0333 | 2 |
| CDC42 | cell division cycle 42 | -1,416 | 0,0047 | 2 |
| ING1 | inhibitor of growth family member 1 | -1,335 | 0,0058 | 2 |
| NMT1 | N-myristoyltransferase 1 | -1,443 | 0,0081 | 2 |
| PCDH7 | protocadherin 7 | -1,245 | 0,0261 | 2 |
| RALGAPB | Ral GTPase activating protein non-catalytic beta subunit | -1,245 | 0,0414 | 2 |
| TARDBP | TAR DNA binding protein | -1,3 | 0,0495 | 2 |
| TNPO2 | transportin 2 | -1,325 | 0,0338 | 2 |
| UQCC1 | ubiquinol-cytochrome c reductase complex assembly factor 1 | -1,517 | 0,0119 | 2 |

**
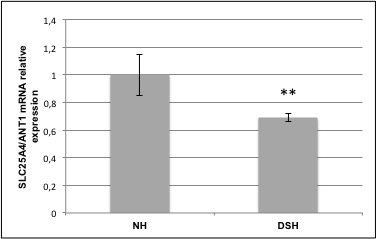
**

**Figure 1. *SLC25A4/ANT1* expression in heart tissue by qRT-PCR.**

Real-time PCR of SLC25A4/ANT1 in trisomic hearts confirmed the downregulation obtained by microarray analysis. Results are expressed as relative mean values ± SEM of 3 trisomic samples (DSH), compared with control hearts (NH) set equal to 1.

∗∗ p <0.01. P-value expresses statistical significance for trisomic versus non trisomic sample comparisons.
