# Supplementary figures and images for: Overexpression of Chromosome 21 miRNAs May Affect Mitochondrial Function in the Hearts of Down Syndrome Fetuses
Source: Int J Genomics. 2017 Sep 5;2017:8737649. doi: 10.1155/2017/8737649 (PMC5605795; doi:10.1155/2017/8737649)

## Slide 1
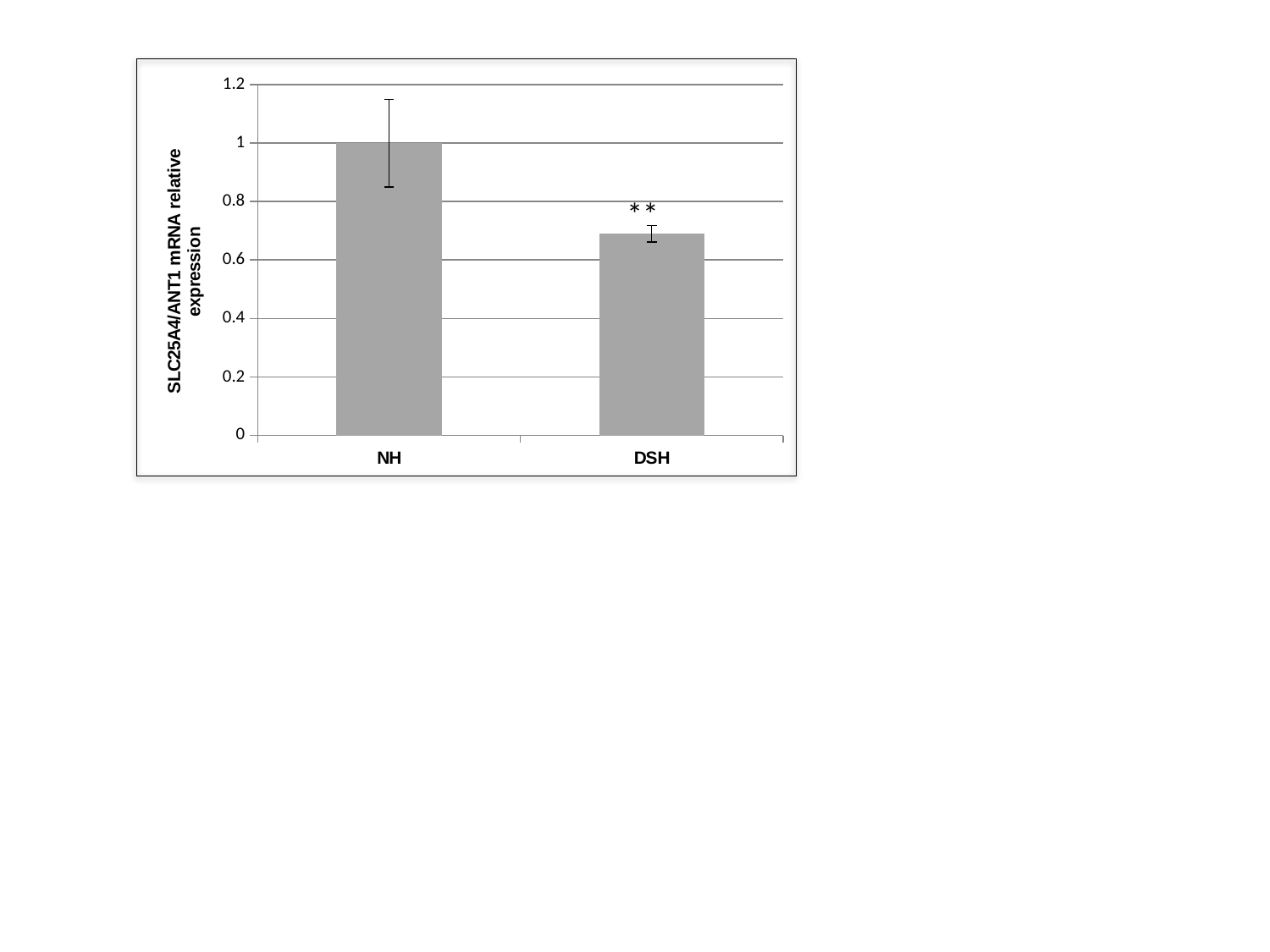

### Chart
| Category | |
|---|---|
| NH | 1.0 |
| DSH | 0.69 |

Supplement: Supplementary file 3 [file 8737649.f3.pptx]
